# Double megatherapy and autologous bone marrow transplantation for advanced neuroblastoma: the LMCE2 study.

**DOI:** 10.1038/bjc.1993.21

**Published:** 1993-01

**Authors:** T. Philip, R. Ladenstein, J. M. Zucker, R. Pinkerton, E. Bouffet, D. Louis, W. Siegert, J. L. Bernard, D. Frappaz, C. Coze

**Affiliations:** Centre Léon Bérard, Pediatric and Bone Marrow Transplant Department, Lyon, France.

## Abstract

In the LMCE1 study using a single course of megatherapy most of the relapses occurred during the first 2 years after autologous bone marrow transplantation. A second pilot study (LMCE2) was therefore set up using a double harvest/double graft approach with two different megatherapy regimens. Objectives were to test the role of increased dose intensity on response status, relapse pattern and overall survival. Thirty-three patients (20 boys, 13 girls) with a median age of 53 months at first megatherapy (range, 17-202 months) entered this study. They were cases either with refractory disease in partial response after second line treatment for stage 4 neuroblastoma (n = 25) or after relapse from stage 4 (n = 5) or stage 3 disease (n = 3). All patients received Etoposid and/or Cisplatinum (or Carboplatin) containing treatments before megatherapy. The first megatherapy regimen was a combination of Tenoposid, Carmustine and Cisplatinum (or Carboplatin), the second applied Vincristin, Melphalan and Total Body Irradiation. The first harvest was scheduled 4 weeks after the last chemotherapy, the second 60 to 90 days after megatherapy. All marrows were purged in vitro by an immunomagnetic technique. Median follow up time since first megatherapy is 56 months. Response rates for evaluable patients were 65% (complete response rate: 16%) for megatherapy 1 and 60% (complete response rate: 25%) for megatherapy 2. Considering that only patients with delayed response or relapse were eligible for this pilot study the overall survival was encouraging with 36% at 2 years and still 32% at 5 years. The costs for these survival rates were high in terms of morbidity (four early and four late toxic deaths; toxic death rate: 24%). Double harvesting may have the disadvantage of delayed engraftments related in part to a disturbance of marrow microenvironment by megatherapy 1. This double megatherapy approach achieved a prolonged relapse free interval (median 11 months, range 2-31 months) in patients reaching megatherapy 2 and justifies further evaluation of concepts with consecutive dose-escalation.


					
Br. J. Cancer (1993), 67, 119-127                                                                     (?) Macmillan Press Ltd., 1993

Double megatherapy and autologous bone marrow transplantation for
advanced neuroblastoma:the LMCE2 study

T. Philip', R. Ladenstein2, J.M. Zucker3, R. Pinkerton4, E. Bouffet', D. Louis5, W. Siegert6, J.L.
Bernard7, D. Frappaz8, C. Coze7, M. Wyss9, D. Beck'0, G. Soulliet5, J. Michon3, I. Philip', F.
Chauvin2, M. Favrot' &         P. Biron'

'Centre Leon Berard, Pediatric and Bone Marrow Transplant Department, 28, rue Laennec, 69373 Lyon Cedex 08, France;

2Centre Leon Berard, Biostatistics Unit- 28, rue Lainnec, 69373 Lyon Cedex 08, France; 31nstitut Curie, Pediatric Department, 26,
rue d'Ulm, 75005 Paris, France; 4Royal Marsden Hospital, Pediatric Department, Down Road, Sutton SM2 SPT Surrey, UK;
5Hopital Debrousse, Pediatric Department, 26 rue des S. Bouvier, 69005 Lyon, France; 6Universitfitsklinikum Charlottenburg,
Hematology Department, Spandauer Damm 130, 1000 Berlin 19, Germany; 7Hopital Nord, Pediatric Department, Boulevard

Dramard, 13075 Marseille, France; 8H6pital Nord, Pediatric Department, Av. A. Raimond, 42277 Saint Priest en Jarez, France;
9H6pital Cantonal de Geneve, Pediatric Department, 30 bd Cluse, 1211 Geneve, Switzerland; `Centre Hospitalo-Universitaire de
Lausanne, Pediatric Department, 1011 Lausanne, Switzerland.

Summary In the LMCEI study using a single course of megatherapy most of the relapses occurred during
the first 2 years after autologous bone marrow transplantation. A second pilot study (LMCE2) was therefore
set up using a double harvest/double graft approach with two different megatherapy regimens. Objectives were
to test the role of increased dose intensity on response status, relapse pattern and overall survival.

Thirty-three patients (20 boys, 13 girls) with a median age of 53 months at first megatherapy (range, 17-202
months) entered this study. They were cases either with refractory disease in partial response after second line
treatment for stage 4 neuroblastoma (n = 25) or after relapse from stage 4 (n = 5) or stage 3 disease (n = 3).

All patients received Etoposid and/or Cisplatinum (or Carboplatin) containing treatments before
megatherapy. The first megatherapy regimen was a combination of Tenoposid, Carmustine and Cisplatinum
(or Carboplatin), the second applied Vincristin, Melphalan and Total Body Irradiation. The first harvest was
scheduled 4 weeks after the last chemotherapy, the second 60 to 90 days after megatherapy. All marrows were
purged in vitro by an immunomagnetic technique. Median follow up time since first megatherapy is 56 months.

Response rates for evaluable patients were 65% (complete response rate: 16%) for megatherapy I and 60%
(complete response rate:25%) for megatherapy 2. Considering that only patients with delayed response or
relapse were eligible for this pilot study the overall survival was encouraging with 36% at 2 years and still
32% at 5 years. The costs for these survival rates were high in terms of morbidity (four early and four late
toxic deaths; toxic death rate:24%). Double harvesting may have the disadvantage of delayed engraftments
related in part to a disturbance of marrow microenvironment by megatherapy 1. This double megatherapy
approach achieved a prolonged relapse free interval (median 11 months, range 2-31 months) in patients
reaching megatherapy 2 and justifies further evaluation of concepts with consecutive dose-escalation.

Stage 4 Neuroblastoma in children over one year of age at
diagnosis is the most common childhood malignancy before
the age of five (Carlsen et al., 1986). Historical groups only
had a survival expectancy of about 10% at 3 years with
conventional chemotherapy (Shafford et al., 1984; Hartmann
et al., 1983; Letourneau et al., 1985; Rosen et al., 1984).
More intensive induction treatments (Shafford et al., 1984;
Bernard et al., 1987, Philip et al., 1987b; Hartmann et al.,
1988; Berthold et al., 1990; Pinkerton et al., 1990; Kushner et
al., 1987; Philip et al., 1988) are now able to achieve initial
response rates up to 90%. Megatherapy (MGT) and other
consolidation modalities further contributed to improve sur-
vival rates (Pritchard et al., 1982; Graham-Pole et al., 1984;
August et al., 1984; D'Angio et al., 1985; Pritchard et al.,
1986; Pinkerton et al., 1987; Graham-Pole 1991a; Dini et al.,
1991; Zucker et al., 1991; Philip et al., 1985, Philip et al.,
1987a; Philip et al., 1991; Hartmann et al., 1986; Hartmann
et al., 1987, Seeger et al., 1991). Although survival at 2 years

has clearly improved to approximately 40%, the long-term
survival remains unlikely for at least 75% of the patients due
to later disease progressions or relapse. (Philip et al., 1991).

Our group has previously reported an intensified induction
treatment (Bernard et al., 1987) and consolidation with Vin-
cristin (VCR), Melphalan (L-PAM) and Total Body Irradia-
tion (TBI) followed by purged Autologous Bone Marrow
Transplantation (ABMT) in 72 consecutive unselected
patients with stage 4 neuroblastoma over one year of age at
diagnosis (Philip et al., 1987a; Philip et al., 1991). A signi-
ficant impact on progression-free survival at 2 years was
achieved, but it is not possible to differentiate if this overall
improvement is related to intensified induction or MGT fol-
lowed by BMT or both. No relapses were observed before 4
months post-MGT in the LMCE1 study (Philip et al., 1991)
with the majority occurring during the following two years.

These results served as the background to a pilot study in
a group of patients who were not eligible for the LMCE1
study (Bernard et al., 1987; Philip et al., 1987a; Philip et al.,
1991). These patients showed delayed response to second or
third line induction treatments or entered the study after
relapse. In an attempt to further improve response and to
decrease the incidence of early relapse, patients received two
different courses of MGT within a 3 months interval. Bone
marrow was harvested twice, before and after the first MGT
(MGT1). The objectives of this study were to assess the effect
of increased dose intensity on response as well as on relapse
pattern and overall survival.

Correspondence: T. Philip, Centre Leon B6rard, 28 rue Laennec,
69373 Lyon Cedex 08, France.

Received 6 May 1992; and in revised form 10 August 1992.

Br. J. Cancer (1993), 67, 119-127

'?" Macmillan Press Ltd., 1993

120     T. PHILIP et al.

Patients and methods
Methods

Parents of all children gave informed consent according to
the Laws for clinical research in France or other countries.
The LMCE2 protocol was reviewed and approved by the
Comite d'Ethique de l'Universite Claude Bernard at Lyon,
France. Patients for this phase II study were selected on the
basis of an estimated poorer outcome due to initial minor
response to multicenter protocols or previous treatment
failure. This study population is heterogenous due to patients
inclusion criteria and bias of case selection cannot be exc-
luded since this is a non-randomised study with a high
percentage of transferred patients.

Definition of disease status

Patient evaluation at diagnosis was heterogenous since
patients were not included in a common protocol at treat-
ment start. Primary tumours were evaluated with CT-scans
and some with ultrasound in addition. Assessment of meta-
stases included iliac bone aspirates (one to four sites) and
core biopsies (one to four sites). At diagnosis applied diag-
nostic methods for evaluation of bone disease were
heterogenous. TC99-scan only was used in 12 patients, mIBG
in eight, both in 11 patients and two patients showed bone
lesions on radiographs (but had no further evaluation). Stan-
darised cytohistology was performed in all patients.

At time of inclusion in the protocol, patients were
uniformly reevaluated in all centers by a full diagnostic work
up regarding residual tumour size, urinary catecholamine
excretion, bone lesions and bone marrow infiltration.
Primary tumour sites and other residual soft tissue masses
were evaluated by CT-scans and ultrasound. As a rule, four
bone marrow aspirates and four trephine biopsies were per-
formed at inclusion, 1 to 2 weeks before MGT and 2 to 3
months after MGT. Bone marrows were evaluated by
immunohistomorphology but a result of positive immunology
was taken into account only when clumps of tumour cells
were diagnosed. Assessment of bone disease was performed
with mIBG-scans in 29 patients (88%) and 14 had additional
Tc99-scans. Four patients had Tc99 scans only. Residual
improved, but positive Tc99 lesions were biopsied to rule out
active disease. Partial response (PR) was defined as pre-
viously published in detail (Philip et al., 1987c) as 50% or
more improvement of at least two criteria (initial tumour,
bone marrow and bone). Sensitive relapse (SR) refers to the
same PR criteria as above. Only one SR in 2nd complete
response (CR) is included in this series. Resistant relapse
(RR) defines minor response (< 50%) or disease progression.
Patients were evaluated for response 2 weeks before MGT1,
60 to 90 days post MGTI and before the second harvest.
Final evaluation was scheduled 2 months after MGT2 and
every 6 months during the first 2 years (then as indicated
clinically).

Bone marrow harvest

Autologous bone marrows were evaluated according to stan-
dards as previously published (Combaret et al., 1989a) using
both cytological and double fluorescence analysis of the bone
marrow. Both bone marrow harvests were treated ex-vivo by
the immunomagnetic depletion procedure as previously pub-
lished in detail (Combaret et al., 1989b; Favrot et al., 1987,
1989) with no detectable residual tumour cells after the pro-
cedure (detection limit 10' tumour cells). Patients receiving

allogenic BMT are not included in this analysis.
Treatments

First line chemotherapy is the original treatment protocol
which the patients entered at diagnosis. Second line
chemotherapy refers to the change in treatment policy due to
minor response to first line treatment. Surgical attempts to

remove the primary tumour were undertaken in all but one
patient. Results of surgery as part of pre-MGT treatments
are given in detail on Table I. In fact, complete excision of
primary tumours was achieved (PT1/PT3A) in only 8 patients
of the PR group. For relapse patients results of surgery are
detailed for first line treatments and was performed only in
five during second line treatment. Three patients had received
additional local radiotherapy before entering the LMCE2
study. A summary is given in Table I.

Pretreatments duration of < 12 months was observed in
17 patients and included one stage 4 relapse patient not
previously grafted. Sixteen patients had longer intervals
between diagnosis and date of entry in the double graft
programm. Nine of them were patients that never had
achieved complete remission and their pretreatments varied
from 12 to 20 months. Seven patients in relapse had intervals
from 15 to 52 months (exact relapse free interval not
reported).

Following inclusion, two courses of Etoposid (VP16) and
Cisplatinum  (CDDP) (n = 10) or VP16 and Carboplatin
(CBDCA) (n = 8) were given to increase response. Fifteen
patients had pretreatment with other CDDP containing com-
binations. As shown in Table II, a first harvest was scheduled
4 weeks after the last chemotherapy. The first MGT regimen
(MGTI), was a combination of Tenoposid (VM26), Bichlo-
ronitrosourea (BCNU) and CDDP or CBDCA and was
given within 2 weeks after harvest. A second harvest was
performed 60 to 90 days post-MGTI. The second MGT
regimen (MGT2) was a combination of VCR, L-PAM and
TBI (Philip et al., 1987a) (Table II). Seven of the 33 patients
had received granulomacrophage colony stimulating factor
(GMCSF) either at MGT1 (one patient or MGT2 (6
patients) as part of a randomised study of this agent (Philip
et al., 1992).

Statistical methods

Probability of survival was analysed according to the
Kaplan-Meier Method (Kaplan & Meier, 1958) regarding the
date of first ABMT as day 0 for survival estimation. All
events of death (toxicity or disease related) were included for
survival estimations. The median follow up time is 70 months
since diagnosis and is 56 months after MGT1.

Patients

Thirty-three patients were included in this LMCE2 pilot
study from 04/85 to 08/88. Twenty were males and 13
females with a median age of 53 months (range: 17-202
months) before first graft.

A total of 25 patients with stage 4 neuroblastoma at
diagnosis were in PR after at least second line induction
treatments. Thirteen patients showed only minor response to
first line protocols and were consequently improved to PR
status with courses of VP16 and high dose CDDP or
CBDCA as previously reported (Philip et al., 1987b; Hart-
mann et al., 1988; Philip et al., 1988). None of these patients
were eligible for the LMCE1 study in which a single MGT
regimen is given. Twelve patients received pretreatments out-
side France: Germany (four cases), Israel (two cases), Italy
(two cases), Switzerland (two cases), USA (one case), Eng-
land (one case) and joined the LMCE2 protocol also in
delayed PR after more than first line treatments.

Nineteen of 25 had bone marrow involvement at the time
of the first harvest and the first MGT. Fourteen had bone

involvement, 7/23 had elevated catecholamine secretion (two
patients not tested) and 14 had primary, locoregional residual
tumour (patient no 9 showed a local progression just before
MGT).

Eight patients were included in the LMCE2 protocol after
relapse from stage 4 (five patients) or stage 3 disease (three
patients). Seven patients were still sensitive to the VP16-
CDDP rescue regimen as previously described (Philip et al.,
1987b) and were defined as SR and received MGT1 in sub-

DOUBLE MGT/ABMT FOR ADVANCED NEUROBLASTOMA (LMCE2 STUDY)  121

4 04 En ) A4 En U) A4 04 V 0 U  0 P 4 a, P r u Z C, 44 o g  O UQ   r 0 V  V  V

0 N       0 10 W t-0 N  o en o CN  -  0 N rn W) W  o o X   o   o o   W Q   u
M N N -N N NZ mN - N .- __e< _N Z Z N-- _  - __tZ ZZ Rt

0 -   0%  r , _  <5  0  %0N _  as m 0   0   0  0  0 o   o o

N No N_nZ   Z_N N _0 -_cZZ \- o

st X  st  e  oo  %oO   tCC)  l0 tn  %t r-  xo  t- ? o  a.,

co Z C O_  N Z Z C-4 4--4 -  Z-- ""-N-4  -.  N

t  0  Os It--  "%o  (  onoR tn m  m   m W  W "o  _

: ~   ~~~~~~~~     Z

Z I     I I +I+I+ IZ+I  I   I+ ++I II
lI IIl++++I+++I+I+I l+++++l
++++++++III++1+1+II++III
++++I+++ I+++++I+I+++ I++ I+

00  0 000004  g   g g ~  0 0   0 0  00 0 0   0 00 0  000000g

< mu.< muu<m.<Um mmumm

QQQ UQ  Q U QQU QQQQQUUV UQU UU

0 -N   e  t  u  %0  r -  00 O   Cl  e  't   t

r-o            i -  - r-

,- t- 00 W0

Z I  I  I  I  I  I I

rY X  XXA

111111+1

0) 0   0 0 0 0 0 0 0 0 U )

u0  I m 0 0  0 -  Cl

l Cl Cl C en rnene

_

k
0t

0S

0

tV

0
.

0

,-0

0

00
Cd
0
'5

'0
co

0
0~

w
0
D

._

CA

=1

.S

Eu

122    T. PHILIP et al.

Table H LMCE 2 protocol in advanced neuroblastoma

-B.M. Harvest I + Purge

BCNU
VM26
CDDP

or

CBDCA

300 mgm-2
250mgm-2
200 mg m-2
250 mgm-2

Day I

Day 2-4
Day 2-4

ABMT 1

Day 2-6

-B.M. Harvest 2+ Purge

VCR          1,5mgm-2 IV Bolus Day 1

0,5mgm-2 CI        Day 1-5   ABMT 2
TBI          12 Gy/6 fractionsa  Day 2-4
Melphalan    180mg m-2          Day 6

aLung protection at 10 Gy B.M. = Bone Marrow; IV = Intra-
Venous; CI = Continuous Infusion; TBI = Total Body Irradia-
tion; VCR = Vincristine; CBDCA = Carboplatin; CDDP = Cisp-
latinum; BCNU = Bichloronitrosourea; VM 26 = Teniposid;
ABMT = Autologous Bone Marrow Transplantation.

sequent CR (one patient) or PR (six patients). One patient
was grafted when progressing on rescue protocol (with VP16
and CDDP) and was defined as a RR. In this group 1/8 had
bone marrow involvement at the time of the first harvest and
before MGT1, none had bone involvement, 0/7 showed on-
going catecholamine secretion (one patient not tested) and
6/8 locoregional residual tumour.

Biological criteria at diagnosis are not available (N-myc,
NSE) in this group of patients or just for a very limited
number (Ferritin and LDH was evaluated in five and three
patients respectively and was not done in all the others).

Twenty-four patients were grafted in Lyon, nine patients in
other centers (in France:3 at the Institut Curie/Paris, one at
Hopital Nord/St Priest en Jarez, one at Hopital Timone/
Marseille; in Germany:two at the Universitatsklinikum
Charlottenburg/Berlin; in Switzerland: one at Hopital
Cantonal/Geneve, one at the Centre Hospitalo-Universitaire/
Lausanne).

Results

First harvest and.first ABMT

At harvest the median number of nucleated cells was
3.02 x 108 kg-' (0.35-10.5 x 0Ikg1-) and the median num-

ber of colony forming units (CFU) was 18 x 104 kg-'
(1.1-130 x 104 kg-'). Reinfused, purged bone marrow con-
tained a median of 0.65 x 108 nucleated cells kg-'

(0.29-1.96 x 108 kg-') and a median of 3 x 104 CFU kg-'

(0.2 to 14 x 104 kg-').

Thirty-one of 33 patients were evaluable for response to
BCNU, VM26 and Platinum as shown in Table I; (two
patients were not evaluable for response:one patient was in
second CR before MGT1 and stayed in CR, and one PR
patient died on day 14 post ABMT1 due to septicaemia). Of
the 31 evaluable patients, five achieved CR and 15 stayed in
PR but showed a reduction in their disease of 50% or more.
Thus the response rate to MGT1 is 65% (CR rate: 16%).
Eight patients had stable disease and three patients had
disease progression. Eight of the 20 positive marrows prior to
MGT1 were normalised by MGT1. Residual tumour was
observed in the bone in eight patients and locoregionally at
the primary tumour site in nine.

Median time to reach 1000 white cells after ABMT1 was
18 days (0-35) and 19 days (0-31) to reach 200 polymor-
phonuclear cells (PNC), 23 days (13-60) to reach 500 PNC
and 22 days (10-78) to reach 50,000 platelets. One toxic
death was observed (disseminated cryptococcus) and one
patient had significant morbidity (endocarditis). Median time
to return home was 30 days post ABMT1.

Second harvest and second ABMT

A second bone marrow harvest was performed 60-90 days
post MGTl and 12 still contained tumour cells (before purg-
ing). At harvest, the median number of nucleated cells was
4.53 x 108 kg-' (0.95-10.6 x 108 kg-') and the median
number of CFU was 24 x I04 kg-' (4.1-75 x I04 kg-'). The
median number of nucleated cells and CFU grafted were
respectively 0.92 x 108 kg-' (0.22-2.92 x 108 kg-') and
3.4 x 104 kg-' (0.3 to 12 x 104 kg-').

Patients characteristics before MGT2, marrow recovery
and response to MGT2 are summarised in Table III. Thirty
of the 33 patients reached MGT2:one patient died with
toxicity after the first graft, two patients with disease progres-
sion after the first graft received no MGT2 (one due to rapid
death with disease, the other was a parental refusal). Median
time to reach MGT2 was 3 months (range, 2-6 months) post
MGT1. Five patients had achieved CR with MGT1, two
additional patients with only residual local disease achieved
CR before MGT2 with surgery (patient no. 31 was in 2nd
CR before MGT1 and is not evaluated for response). One
patient (patient no 7) died of toxicity and was not evaluable
after the second graft, patient no 25 was not evaluated. Thus
only 20 of the 30 patients are evaluable for the response to
VCR, L-PAM and TBI. Five achieved CR and seven patients
were further improved by 50% or more (PR). The response
rate of these 20 patients to MGT2 is thus 60% (CR
rate:25%).

Duration of aplasia was longer after MGT2 and ABMT2.
The median time to reach 1000 white cells was 30 days
(12-120), to reach 200 PNC cells 25 days (10-124), to reach
500 PNC cells 43 days (15-120). Twelve patients reached
50,000 platelets at a median time of 65 days (27-297). Six
patients died with procedure related complications before
reaching 50,000 platelets and in 12 patients the date of
recovery was not reported due to their return to their trans-
ferring centers. Two patients (no. 1 and no. 27) with delayed
platelets recovery died with disease. One never became
independent of platelets transfusions since he experienced
early relapse 3 months after MGT2 and died with progres-
sion 4 months later. The other one (no. 1) became indepen-
dent after 120 days but died with disease progression one
year after MGT2. Two further patients (no. 2 and no. 25)
with delayed platelets recovery are alive and well after a
prolonged period of platelet substitution dependency
(reaching stable counts of more than 50,000 at days 189 and
297). The median time to return home was 40 days.

Three deaths due to interstitial pneumonitis occurred
within the first 100 post-transplant days in two CR patients
(days 35 and 78) and in one PR patient (day 41). Four
further late toxic deaths were observed due to haemorrhage
associated with delayed platelet recovery (one PR
patient:days 118 and one CR patient:day 121), lung fibrosis
in a PR patient on day 138 and renal failure with incomplete
marrow engraftment in a PR patient on day 180.

Late and long term complications in the 11 survivors were
documented growth impairment in one and hemosiderosis in
another. The other patients appear to have normal life
quality comparable to their age companions with full rein-
tegration in their social structure.

Survival

The overall survival for the 33 patients after a median follow
up time of 56 months is 36% (Figure 1) at 2 years and shows

an actuarial 32% plateau at 5 years. Survival of patients was
not influenced by the interval between diagnosis and study
entry since actuarial survivals are exactly the same for
patients with intervals of less or more than 12 months.

Twenty-five patients receiving MGT1/2 in first partial
remission after second line treatments (with previous minor
response to first line treatment) achieved an overall survival
of 40% at 2 years and an actuarial survival of 34%  at 5
years. Eight patients treated with MGT1/2 for relapse (one
second CR, 6 second PR and one minor response) had an
overall survival of 25% at 2 years (5 years not reached)
(Figure 2).

DOUBLE MGT/ABMT FOR ADVANCED NEUROBLASTOMA (LMCE2 STUDY)  123

00
1-

-C

'0

00

en
-'

a;

oo
0-

t-

1-

esi
CU4
'-

co

10

UU ZCACnz2 gzUaQ4 A4UA4 u z  CL4u.Cuuuzu

>X >ZZtZZ"ZZZZ"Z   ZZo  0gnZzzzzzz

tl A  oD t _ qt (D en  tn "    WI - , n _ ) 1^ ON <D
en> Z 'S " " m Z m "D 4 -t -n Z Z _ el m ,t " t -

o0 N W t- , t-V tn C W  o " C7 9 t- -e t- a o W)  $ z

m " Z " "-       Z llqt M MZ    - -   ->     _

I   I   I   I   I  I   I   I   I  +   I   I   I   I   I   I   I   I   I
lI I I I +++ I I I + I I + I I     I I ++ I
+++++    +   I I I I I + I I I + I + I I I I
I + + + I + + + + I + + + +II I I II I +

0D   ".  m   mt   W)   4.-  00 C)M 11'W

<D O      wi I 7m 7t

ZO%Z Z

+  I  +  I  I  I  I  I

W)  as W V)VQ

U      U o U U U

t0 4-  000  0s Q   "- .   M

q)
04 Z

'F-

00a

oo~~~~~~u

A4 1

;^~~~~~~~~~~~~~~~~1r

E-P

'20

o

.-

CU4

O     cU

0
'0

CU CCU

20
wq >
'0

L._

00

u

>D 0

CU0   '

0  _)  U LE

CU
CU4  it

oO 0 a 4)
3'CU CC

E 4)E-

*-~  C,  CU

,0_--,

-

-
4 _

0
CU

-

0

CU

I4)

Ca

0

CU

0

4-

0

t

Cd

C-

0

CU

0

4)

CU

'0

to

CU

0
04

0

64)

U

la

0

4)

4.4

I

124     T. PHILIP et al.

Table IV Sites of disease progression after autologous bone marrow transplantation

Patient's Primary  Status      Duration of Progression  Site of Progression  Cause of Death
Number   Tumour   after           Free interval post   after ABMT 2         or last Disease

Site     ABMT 2        ABMTJ      ABMT2                                Status

(in months)
(1) Grafted in PR:

I       n.i.     CR            16           13     Bone Marrow, Skeleton         Disease
4       Adrenal SD              8            6     Bone Marrow, Skeleton,         Disease

Mediastinum, Lungs, Liver

5       Adrenal SD             19           15     Retroperitoneum                Disease
6       Retrop.  SD             11           7     Bone Marrow, Skeleton,         Disease

Retroperitoneum

12       Adrenal PR             12           10     Liver, Skeleton               Disease
13       Adrenal PD              6            3     Bone Marrow, Skeleton         Disease

16       Adrenal PD              3            1     Retroperitoneum               Disease and

Haemorrage
17       Retrop.  CR            13           9      Lungs                         Disease
18       Adrenal PR             17           13     Bone Marrow, Skeleton,        Disease

Retroperitoneum

*19      Adrenal PD               3                 Retroperitoneum, Mediastinum   Disease
*22      Adrenal PD               2                 Bone Marrow, Skeleton,         Disease

Retroperitoneum,

Central Nervous System
(2) Grafted in relapse:

26       Adrenal PR               8           3     Mediastinum                    Disease
27       Adrenal CR               6           3     Mediastinum                    Disease
29       Retrop.   CR             9           6     Retroperitoneum                Disease

31       Adrenal CR              31          26     Skeleton                       Alive in relapse

a = No second graft; CR = Complete Response; PR = Partial Response; SD = Stable Disease; PD = Progres-
sive  Disease;  ABMT = Autologous     Bone   Marrow    Transplantation.  n.i. = not  identified;  Ret-
rop. = Retroperitoneum.

L-

Co

Eco

5L)
0

20
10

, \

13       26        39        52

Months from first graft

Figure 1 Overall survival of 33 study patients.

Fifteen patients experienced relapse or disease progression.
Two patients had very early metastatic and locoregional
relapses at 2 and 3 months after MGT1 and the other 13
after MGT2. The sites of relapse or progression are detailed
on Table IV. Nine patients showed distant relapse (two
N = 33 P= 33%     tumours recurred also in the primary site) whereas in three

patients primary locoregional tumour was the first site of
recurrent disease. The median duration of the progression
free interval was 11 months (range, 2 to 31 months) post
MGT1 for the 13 patients. Eight patients died from treat-
ment related toxicity. Thus there are ten out of 33 patients
surviving relapse-free 32 to 77 months after MGTI and seven
65     78         of them more than 4 years at the time of this report.

Discussion

-Relapse N = 8 P = 25%
--- PR   N=25P =36%

13       26       39       52

Months from first graft

65      78

Figure 2 Survival of the 25 patients in PR and eight patients
in relapse (seven SR, one RR).

MGT followed by ABMT as consolidation treatment in
neuroblastoma has been investigated by several teams in very
different study populations, treating either only patients in
first CR after induction or in addition patients with residual
locoregional disease and/or patients in PR after one line of
induction treatment (Pritchard et al., 1982; Hartmann et al.,
1986; Philip et al., 1991; Graham-Pole et al., 1991a; Dini et
al., 1991) but rarely patients with less sensitive disease with
two or more lines of induction therapy.

We report on a subgroup of stage 4 neuroblastoma
patients who only achieved delayed PR after at least second
line treatments or had relapsed. All received consolidation
treatment that consisted of two different MGT regimens
(BCNU, VM26, CDDP/or CBDCA and VCR, L-PAM,
TBI), given within a 3 months interval, each one preceded by
harvesting and purging of the bone marrow.

This patient cohort fulfilled in our view poor prognosis
criteria at the time of inclusion. In the group of PR patients,
19 (76%) still had bone marrow involvement at first harvest
after intensive pretreatments and 14 (56%) still had active

loo10

90-
80-
701

L-

Co

Co
a)

0

60
50
40
30
20
10

0

u

...                      .

,l
1001

90 -
80 -
70 -

60 -                         - LMCE2
50

40,,'
301

L

I

L -

IL -

II---

,--  ---- ----
I

DOUBLE MGT/ABMT FOR ADVANCED NEUROBLASTOMA (LMCE2 STUDY)  125

bone disease. In fact only three patients had residual disease
only at their primary tumor site, but one of them eventually
showed local progression just before megatherapy (so in two
of these one could in fact estimate a better prognosis than for
the rest of patients). Further, there are eight relapse patients.
Relapse signalises failure of previous treatments and tumours
have a high chance to hold chemoresistant clones. This is in
line with EBMT registry data showing rapid disease progres-
sion after MGT for most relapse patients. Only relapse
patients in second complete remission have an actuarial sur-
vival of 32% post MGT (manuscript in preparation, unpub-
lished EBMT data). Thus poor prognosis can be assumed
also for the relapse patients, probably with the exception of
one patient achieving CR before MGT who was not excluded
from analysis.

We previously observed in the European Bone Marrow
Transplantation Registry data an advantage for neuro-
blastoma patients with pretreatment durations of more than
12 months. This was found to be related to patients transfer-
ral to major transplant centers. To exclude such a bias we
compared pretreatment durations in the LMCE2 study, but
could not detect any influence on outcome. Nevertheless,
patient transferral to major transplant centers always carries
the risk of case selection and we therefore cannot exclude
such a bias.

The toxicity of the MGT1 was acceptable (i.e. one toxic
death and 30 days median time to return home) and pro-
duced a response rate of 65%. In contrast, the CR rate of
only 16% is inadequate to rely only on this intensification
regimen to increase survival. Moreover two patients showed
progressive disease after MGT1 and did not reach the second
procedure. The second MGT regimen was more effective, as
previously reported for a different group of patients, (Pinker-
ton et al., 1987), achieving a further 60% response rate with
a CR rate of 25%. Complete remission in 11/33 patients
(excluding 2 CR due to subsequent surgery) is hence
encouraging for this patient cohort with either initial minor
response or recurrent disease. Disease evaluation was done in
all patients at certain time points after study entry as out-
lined previously. Undoubtedly, any response data taken at
any time reflects comparison of consecutive evaluations dur-
ing the course of treatment and serves as help to estimate
effects. Additive effects of consecutive treatments cannot be
separated and this has to be taken into account especially
when regarding the additional effects of MGT2 in this series
of patients. Ideally, future double MGT concepts would use
more effective drugs for MGT1 but additive toxicity is the
major problem. Thus different concepts of dose escalations
using repetitive high dose monotherapies with growth factor
support appear as the more interesting approach nowadays.

The probability of survival is 32% at 5 years with a
median follow up time of 56 months. This is an equivalent
outcome to patients entering MGT programmes after first
line treatments and better initial response. However, as
previously shown by various teams, efficacy and toxicity are
not separable and the cost of CR in terms of toxic death was
high in this study.

Concerning the double harvest approach we found that the
second harvest was not a problem in terms of nucleated cells
harvested. The number of GMCFU harvested was also very
similar at first and second harvests. Also the number of
nucleated cells was not different from one harvest to the
other. Thus it does not appear that stem cell depletion due to
toxicity to MGT1 was the cause for delayed engraftment
after MGT2 as demonstrated by the clear delay in the time
to return home, the time to reach 500 PNC cells and the
platelet recovery.

We previously reported that immunomagnetic depletion
was mildly toxic to bone marrow precursors as evaluated by

their in vivo clonogenic efficiency although a substantial loss
of bone marrow cells usually occurs (Combaret et al., 1989b).
The recovery of mononucleated cells after the purging proce-
dure ranges from 32 to 42% in our overall experience with
treated marrows with no difference between first and second
harvests. The potential toxicity on hematopoetic progenitor
cells of the immunomagnetic purging procedure or of the
monoclonal antibodies used to target neuroblastoma cells
(specially Thyl) must be considered. However, results
observed in patients treated with only one MGT and rein-
fusion of ex-vivo treated marrow (Philip et al., 1991; Lopez et
al., 1987; Gee et al., 1987) do not favour such hypothesis.

The feasibility of repeated ABMT with mafosfamid treated
marrow was previously reported as safe and effective for
patients after intensive chemotherapy regimens by Hartmann
and Beaujean (Hartmann et al., 1987; Beaujean et al., 1989).
In contrast, this group had collected a single large marrow
harvest before MGT1. Using this divided harvest approach
observed a much shorter delay in engraftment after the
second ABMT than observed in our study. Thus a possible
explanation for the delay of the second engraftment is
damage to the micro-environment by MGT1. This is in line
with a recent and earlier reports stressing the impact of
treatment intensity on bone marrow recovery (Graham-Pole
et al., 1991b, Kingston et al., 1984). In addition, some of our
LMCE2 patients with very late recovery showed an excess of
CD8-Leu 7 cells and were thus given anti-CD8 monoclonal
antibody. This was an effective treatment in some of these
cases as previously reported (Favrot et al., 1990).

Twenty-one of 33 patients included in this LMCE2 study
and analysed at the time of the first harvest had residual
bone marrow metastases and the question could be asked as
to whether the first course of MGT (BCNU, VM26,
CBDCA) was able to eliminate residual tumour cells in the
bone marrow in vivo. This effect was observed in only eight
of the 20 patients, with twelve patients still showing positive
bone marrows at the second harvest. One patient with nor-
mal bone marrow before MGT1 even progressed with the
appearance of bone marrow metastases after MGT1. In 'vivo
purging' by MGT1 was not particularly efficient and
therefore purging procedures appeared rational for the
second harvest in most patients. Purging is however only of
potential value in those cases where MGT achieves CR as
any theoretical adverse effect of reinfusing tumour cells is
irrelevant where significant marrow disease remains in the
patient.

In summary, this treatment approach achieved an
encouraging survival rate of 36% at 2 years with no late
relapses at the time of this report in a subgroup of poor
prognosis neuroblastoma patients. The delayed engraftments
and the high toxic death rate after the seond graft supports
the use of a single harvest approach. This study suggests that
increased dose-intensity may improve the outlook for some
patients with delayed reponse or relapse and it is the task of
future randomised studies to evaluate the role of such mul-
tiple high dose procedures (Shuster et al., 1991). Modification
of the MGT regimens, the use of marrow growth factors and
adjunctive peripheral blood stem cell transfusion could
reduce morbidity to a more acceptable level.

This work has been supported by the Institut National de la Sante et
de la Recherche Medicale (INSERM reseau LMCE No 48.60.22)
and the Ligue Nationale de Lutte contre le Cancer (Comite du
Rh6ne).

R. Ladenstein is a recipient of the Erwin Schrodinger grant.

The authors thank Jorn Treuner, Bernhard Kremens, Rina Zaizov,

Manuel Castello and John Pritchard for entering patients in this
study. They also thank Zora Abdelbost for excellent secretarial
assistance.

126   T. PHILIP et aL
References

AUGUST, C.S., SEROTA, F.T., KOCH, P.A., BURKEY, E., SCHLES-

INGER, H., ELKINS, W.L., EVANS, A.E. & D'ANGIO, G.J. (1984).
Treatment of advanced neuroblastoma with supralethal
chemotherapy, radiation, and allogenic or autologous marrow
reconstitution. J. Clin. Oncol., 2, 609-616.

BEAUJEAN, F., HARTMANN, O., BENHAMOU, E., LEMERLE, J. &

DUEDARI, N. (1989). Hemopoietic reconstitution after repeated
autologous transplantation with mafosfamide purged marrow.
Bone Marrow Transplant., 4, 537-541.

BERNARD, J.L., PHILIP, T., ZUCKER, J.M., FRAPPAZ, D., ROBERT,

A., MARGUERITTE, G., BOILLETOT, A., PHILIPPE, N., LUTZ, P.,
ROCHE, H. & PINKERTON, R. (1987). Sequential Cispaltinum-
VM 26 and Vincristin-Cyclosphamid-Dorubicin in metastatic
Neuroblastoma. An effective alternating non cross resistant
regimen ? J. Clin. Oncol., 5, 1952-1959.

BERTHOLD, F., BURDACH, S., KREMENS, B., LAMPERT, F.,

NIETHAMMER, D., RIEHM, H., RITTER, J., TREUNER, J., UTSCH,
S. & ZIESCHANG, J. (1990). The role of chemotherapy in the
treatment of children with neuroblastoma stage 4: The GPO
(German Pediatric Oncology Society) experience. Klin. Pddiatr.,
202, 262-269.

CARLSEN, N.L.T., CHRISTENSEN, I.B.J., SCHROEDER, H., BRO, P.V.,

ERICHSEN, G., HAMBORG-PEDERSEN, B., JENSEN, K.B. &
NIELSEN, O.H. (1986). Prognostic factors in neuroblastomas
treated in Denmark from 1943 to 1080. A Statistical estimate of
prognosis based on 253 cases. Cancer, 58, 2726-2735.

COMBARET, V., FAVROT, M.C., KREMENS, B., PHILIP, I., BAILLY,

C., FONTANIERE, B., GENTILHOMME, O., CHAUVIN, F.,
ZUCKER, J.M., BERNARD, J.L. & PHILIP, T. (1989a).
Immunological detection of neuroblastoma cells in bone marrow
harvested for autologous transplantation. Br. J. Cancer, 59,
844-847.

COMBARET, V., FAVROT, M., PHILIP, I., CHAUVIN, F., COZE, C.,

CLAPISSON, G., MEZZIANE, F., BERNARD, J.L., ZUCKER, J.M.,
BRUNAT-MENTIGNY, M. & PHILIP, T. (1989b). Autologous bone
marrow transplantation with an immunomagnetically purged
bone marrow in neuroblastoma:analysis of 59 single ADMT and
32 double ABMT. In Autologous Bone Marrows Transplantation,
Proceedings of the 4th International Symposiwn. Dicke, K.A.,
Spitzer, G., Jagannath, S. & Evinger-Hodges, M.J. (eds) p
549-557, Houston.

D'ANGIO, G.J., AUGUST, C., ELKINS, W., EVANS, A.E., SEEGER, R.,

LENARSKY, C., FEIG, S., WELLS, J., RAMSAY, N., KIM, T.,
WOODS, W., KRIVIT, W., STANDJORD, S., COCCIA, P. & NOVAK,
L. (1985). Metastatic neuroblastoma managed by supralethal
therapy and bone marrow reconstitution (BMRc). Results of a
four-institution children's cancer study group pilot study. In
Advances in Neuroblastoma Research. No 1, Evans, A.E., D'An-
gio, G.J., Knudson, A.G. & Seeger, R.C., (eds) p557-564, Alan
R. Liss, Inc; New York.

DINI, G., LANINO, E., GARAVENTA, A., ROGERS, D., DALLORSO, S.,

VISCOLI, C., CASTAGNOLA, E., MANNO, G., BRISIGOTTI, M.,
ROSANDA, C., PASINO, M., RIVABELLA, L., BONI, L., MAR-
CHESE, N., IVANI, G., RIZZO, A., FRANZONE, P., TRASINO, S. &
DE BERNARDI, B. (1991). Myeloblative therapy and unpurged
autologous bone marrow transplantation for poor prognosis
neuroblastoma:report of 34 cases. J. Clin. Oncol., 6, 962-969.
FAVROT, M.C., PHILIP, I., COMBARET, V., MARITAZ, 0. & PHILIP T.

(1987). Experimental evaluation of an immunomagnetic bone
marrow. Purging procedure using a Burkitt lymphoma model.
Bone Marrow Transplant., 2, 59-66.

FAVROT, M.C., COMBARET, V., COZE, C., PHILIP, I. & PHILIP T.

(1989). Is bone marrow purging efficient and necessary for
ABMT in solid tumours? In Bone Marrow Transplanta-
tion:Current Controversies, Liss A.R. (ed), p 289-299.

FAVROT, M.C., PHILIP, T., COMBARET, V., LEMERLE, J., MARIE, J.P.

& BERNARD, A. (1990). Effect of in vivo therapy with a CD8
monoclonal antibody on delayed engraftment after autologous
bone marrow transplantation. Bone Marrow Transplant., 5,
33-38.

GEE, A.P., GRAHAM-POLE, J., LEE, C., BRUCE, K., PICK, T.,

HARVEY, W., WORTHINGTON-WHITE, D., HINTrZ, M., JANSSEN,
W. & GROSS, 5. (1987). Transplantation for neuroblastoma using
immunomagnetically purged autologous bone marrow. In
Autologous Bone Marrow Transplantation, Proceedings of the 3rd
International Symposium. Dicke, K.A., Spitzer, G. & Jagannath,
S., (eds), p 425-431, Houston.

GRAHAM-POLE, J., LAZARUS, H.M., HERZIG, R.H., GROSS, S., COC-

CIA, P., WEINER, R. & STRANDJORD, S. (1984). High-dose Mel-
phalan for the treatment of children with refractory neuroblas-
toma and Ewing's Sarcoma. Am. J. Pediatr. Hematol. Oncol., 6,
17-26.

GRAHAM-POLE, J., CASPER, J., ELFENBEIN, G., GEE, A., JANSSEN,

W., KOCH, P., MARCUS, R., PICK, T., SHUSTER, J., SPRUCE, W.,
THOMAS, P. & YEAGER, A. (1991a). High dose
chemoradiotherapy supported by marrow infusions for advanced
neuroblastoma:a pediatric oncology group study. J. Clin. Oncol.,
9, 152-158.

GRAHAM-POLE, J., GEE, A., EMERSON, S., GALLO, J., LEE, C.,

LUZINS, J., JANSSEN, W.E., PICK, T., WORTHINGTON-WHITE, D.,
ELFENBEIN, G., GROSS, S. & WEINER, R. (1991b). Myeloablative
chemoradiotherapy and autologous bone marrow infusions for
treatment of neuroblastoma:factors influencing engraftment.
Blood, 78, 1607-1014.

HARTMANN, O., SCOPINARO, M., TOURNADE, M.F., SARRAZIN, D.

& LEMERLE, J. (1983). Neuroblastomes traites a lIlnstitut Gus-
tave Roussy de 1975 a 1979, cent soixante-treize cas. Arch. Fr.
Pediatr., 40, 15-21.

HARTMANN, O., KALIFA, C., BENHAMOU, E., PATTE, C.,

FLAMANT, F., JULIEN, C., BEAUJEAN, F. & LEMERLE, J. (1986).
Treatment of advanced neuroblastoma with high dose melphalan
and autologous bone marrow transplantation. Cancer Chemother.
Pharmacol., 16, 165-169.

HARTMANN, O., BENHAMOU, E., BEAUJEAN, F., KALIFA, C.,

LEJARS, O., PATTE, C., BEHARD, C., FLAMANT, F., THYSS, A.,
DEVILLE, A., VANNIER, J.P., PAUTARD-MUCHEMBLE, B. &
LEMERLE, J. (1987). Repeated high-dose chemotherapy followed
by purged autologous bone marrow transplanation as consolida-
tion therapy in metastatic neuroblastoma. J. Clin. Oncol., 5,
1205-1211.

HARTMANN, O., PINKERTON, R., PHILIP, T., ZUCKER, J.M. &

BREATNACH, F. (1988). Very high dose Cisplatin and Etoposid
in children with untreated advanced neuroblastoma. J. Clin.
Oncol., 6, 44-50.

KAPLAN, E. & MEYER, P. (1958). Non parametric estimation from

incomplete observations. J. Am. Statis. Ass., 53, 457-481.

KINGSTON, J.E., MALPAS, J.S., STILLER, C.A., PRITCHARD, J. &

MCELWAIN, T.J. (1984). Autologous bone marrow transplanta-
tion contributes to haemopoietic recovery in children with solid
tumours treated with high dose melphalan. Brit. J. Haematol., 58,
589-595.

KUSHNER, B.H. & HELSON, L. (1987). Coordinated use of sequential

escalted cyclophosphamid and cell-cycle specific chemotherapy
(the N4SE protocol) for advanced neuroblastoma:Experience
with 100 patients. J. Clin. Oncol., 5, 1746-1751.

LETOURNEAU, J.N., BERNARD, J.L., HENDREN, H.W. & CARCAS-

SONNE, M. (1985). Role of surgery in the treatment of neuroblas-
toma. J. Pediatr. Surg., 20, 244-249.

LOPEZ, M., ZUCKER, J.M., URRESOLA, R., DOUAY, L., QUINTANA,

E., KEMSHEAD, J., GORIN, N.C. & VILCOQ, J.R. (1987). Influence
of single and double immunomagnetic depletion on the
hemopoietic capacity or marrow in patients with advanced
neuroblastoma submitted to autologous bone marrow transplan-
tation. Bone Marrow Transplant., 2, 413-419.

PHILIP, T., BERNARD, J.L., ZUCKER, J.M., SOUILLET, G., FAVROT,

M., PHILIP, I., BORDIGONI, P., LUTZ, J.P., PLOUVIER, E., CAR-
TON, P., ROBERT, A. & KEMSHEAD, J.T. (1985). Purged ABMT
in 25 cases of very bad prognosis Neuroblastoma. Lancet, 2,
576-577.

PHILIP, T., BERNARD, J.L., ZUCKER, J.M., PINKERTON, R., LUTZ,

P., BORDIGONI, P., PLOUVIER, E., ROBERT, A., CARTON, R.,
PHILIPPE, N., PHILIP, I., CHAUVIN, F., FAVROT, M. (1987a).
High dose chemoradiotherapy with BMT as consolidation treat-
ment in Neuroblastoma:an unselected group of stage 4 patients
over one year of age. J. Clin. Oncol., 5, 266-271.

PHILIP, T., GHALIE, R., PINKERTON, R., ZUCKER, J.M., BERNARD,

J.L., LEVERGER, G. & HARTMANN, 0. (1987b). A phase II study
of high dose Cisplatin and VP 16 in Neuroblastoma. A report
from the Societe franeaise d'Oncologie Pdiatrique. J. Clin.
Oncol., 5, 941-950.

PHILIP, T., HELSON, L., BERNARD, J.L., ZUCKER, J.M., KREMENS,

B., FAVROT, M.C. & HARTMANN, 0. (1987c). Definition of res-
ponse and remission in children over one year of age with
advanced neuroblastoma. Ped. Hemat. Oncol., 4, 25-31.

DOUBLE MGT/ABMT FOR ADVANCED NEUROBLASTOMA (LMCE2 STUDY)  127

PHILIP, T., GENTET, J.C., CARRIE, C., FARGE, A., MEZIANE, F.,

BOUFFET, E., ZUCKER, J.M. KREMENS, B. & BRUNAT-
MENTIGNY, M. (1988). Phase II studies of combinations of drugs
with high dose Carboplatin in Neuroblastoma (800 mg/m2 to
1.150 g/m2):a report from the LMCE group. In Adances in
Neuroblastoma Research. no 2, Evans, A.E., D'Angio, G.J.,
Knudson, A.G. & Seeger, R.C., (eds) p 573-582, Alan R. Liss,
Inc:New York.

PHILIP, T., ZUCKER, J.M., BERNARD, J.L., LUTZ, P., BORDIGONI, P.,

PLOUVIER, E., ROBERT, A., ROCHE, H., SOUILLET, G., BOUF-
FET, E., MICHON, J., LOPEZ, M., VILCOQ, J.M. GENTET, J.C.,
PHILIP, I., FAVROT, M.C., CHAUVIN, F. (1991). Improved sur-
vival at 2 and 5 years in the LMCE1 unselected group of 72
children with stage 4 neuroblastoma over 1 year of age at diag-
nosis:is cure possible in a small subgroup. J. Clin. Oncol., 9,
1037-1044.

PHILIP, T., MICHON, J., BERNARD, J.L., CHAUVIN, F., STERN, A.,

BOUFFET, E., ZUCKER, J.M. & RAYBAUD, C. (1992). Double-
blind study of safety and efficacy of cho-cell granulocyte-
macrophage colony stimulating factor (GM.CSF) given int-
ravenously for 14 days in patients with neuroblastoma after
autologous bone marrow transplantation compared to int-
ravenous infusion of placebo. In The Comparative Effects of
Recombinant Myeloid Colony Stimulating Factors in Man. Peters,
W.P. (ed), (in press).

PINKERTON, C.R., PHILIP, T., BIRON, P., FRAPPAZ, D., PHILLIPE,

N., ZUCKER, J.M., BERNARD, J.L., PHILIP, I., KEMSHEAD, J. &
FAVROT, M.C. (1987). High-dose Melphalan, Vincristin and total
body irradiation with ABMT in children with relapsed neuroblas-
toma. A phase II study. Med. Ped. Oncol., 15, 236-240.

PINKERTON, C.R., ZUCKER, J.M., HARTMANN, O., PRITCHARD, J.,

BROADBENT, V., MORRIS-JONES, P., BREATNACH, F., CRAFT,
A.E., PEARSON, A.D.J. & WALLENDSZUS, K.R., PHILIP T. (on
behalf of the European Neuroblastoma Study Group, ENSG).
(1990). Short duration, high dose, alternating chemotherapy in
metastatic neuroblastoma. (ENSG 3C induction regimen). Br. J.
Cancer, 62, 319-323.

PRITCHARD, J., McELWAIN, T.J. & GRAHAM-POLE, J. (1982). High

dose melphalan with autologous bone marrow for treatment of
advanced neuroblastoma. Br. J. Cancer, 45, 86-94.

PRITCHARD, J., GERMOND, S., JONES, D., DE KRAKER, J. & LOVE,

S. (1986). Is high dose Melphalan of value in treating advanced
neuroblastoma? Preliminary results of a randomized trial by the
European Neuroblastoma Study Group. Proc. Am. Soc. Clin.
Oncol., 5, 205.

ROSEN, E.M., CASSADY, J.R., FRANTZ, C.N., KRETSCHMAR, C.,

LEVEY, R. & SALLAN, S.E. (1984). Neuroblastoma:The Joint
Center for Radiation Therapy/Dana Farber Cancer Institute/
Children's Hospital Experience. J. Clin. Oncol., 2, 719-732.

SEEGER, R.C., VILLABLANCA, J.G., MATTHAY, K.K., HARRIS, R.,

MOSS, T.H., FEIG, S.A., SELCH, M., RAMSAY, N. & REYNOLDS,
C.P. (1991). Intensive chemotherapy and autologous bone marrow
transplantation for poor prognosis neuroblastoma. In Advances in
Neuroblastoma Research. no 3, Evans. A.E., D'Angio. G.J.
Knudson, A.G. Seeger, R.C., (eds) p 527-533, Alan R. Liss, Inc.
New York.

SHAFFORD, E.A., ROGERS, D.W. & PRITCHARD, J. (1984). Advanced

neuroblastoma:improved response rate using a multiagent
regimen (OPEC) including sequential cisplatin and VM-26. J.
Clin. Oncol., 2, 742-747.

SHUSTER, J.J., CANTOR, A.B., MCWILLIAMS, N., MCWILLIAMS, N.,

POLE, J.G., CASTLEBERRY, R.P., MARCUS, R., PIC, T., SMITH,
E.I. & HAYES, F.A. (1991). The prognostic significance of
autologous bone marrow transplantation in advanced neuroblas-
toma. J. Clin. Oncol., 6, 1045-1049.

ZUCKER, J.M., PHILIP, T., BERNARD, J.L., MICHON, J., BOUFFET,

E., GENTET, J.C., LOPEZ, M., COZE, C., PHILIP, I., BORDIGONI,
P., PLOUVIER, E., MAZINGUE, F., VOLCOQ, J.R. & ASSELAIN, B.
(1991). Single or double consolidation treatment according to
remission status after initial therapy in metastatic neuroblastoma:
first results in LMCE 3 study in 40 patients. In Advances in
Neuroblastoma Research 3, Evans, D'Angio, Knudson, Seeger (eds)
p 543-551, New York, Wiley-Liss, Inc.

				


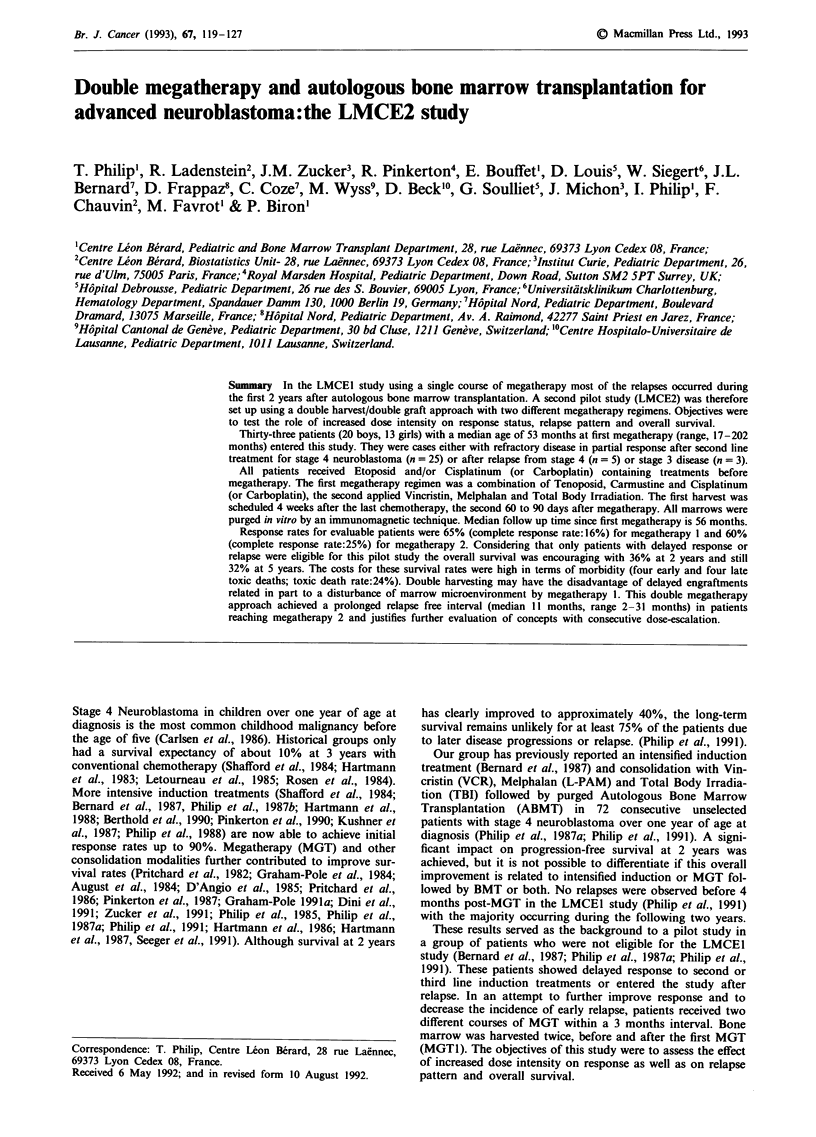

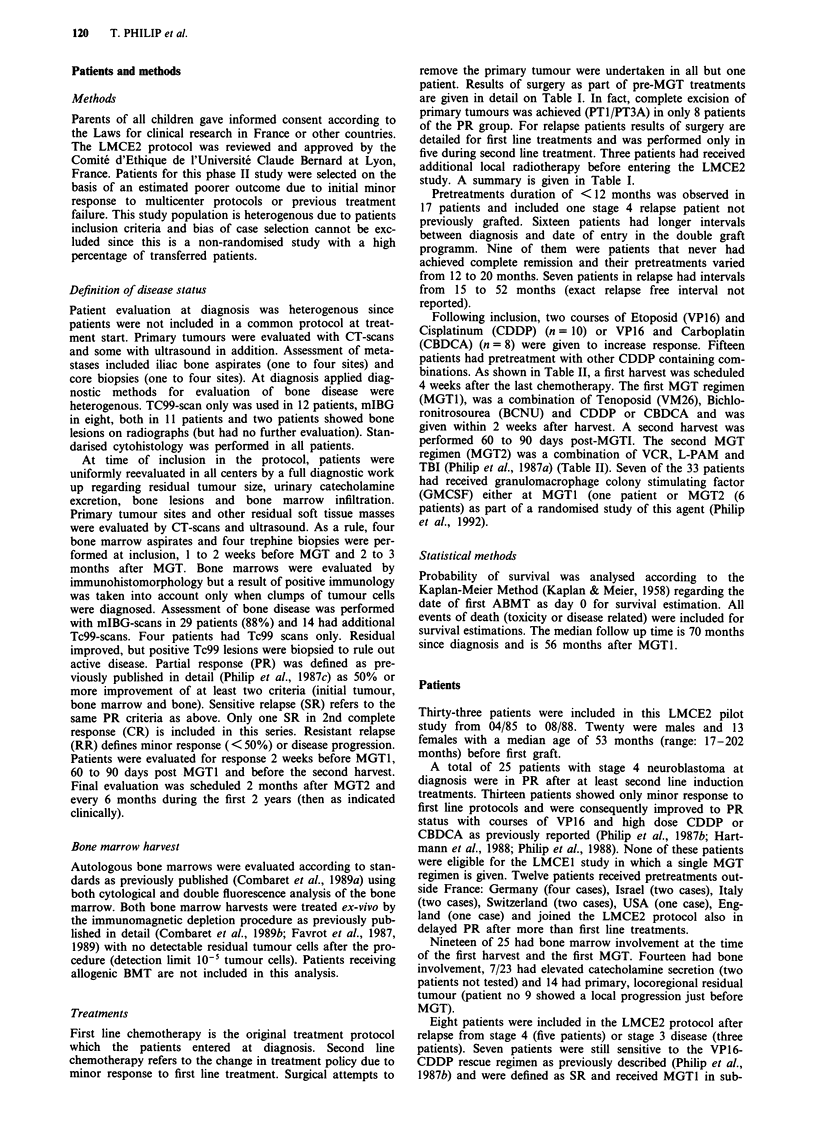

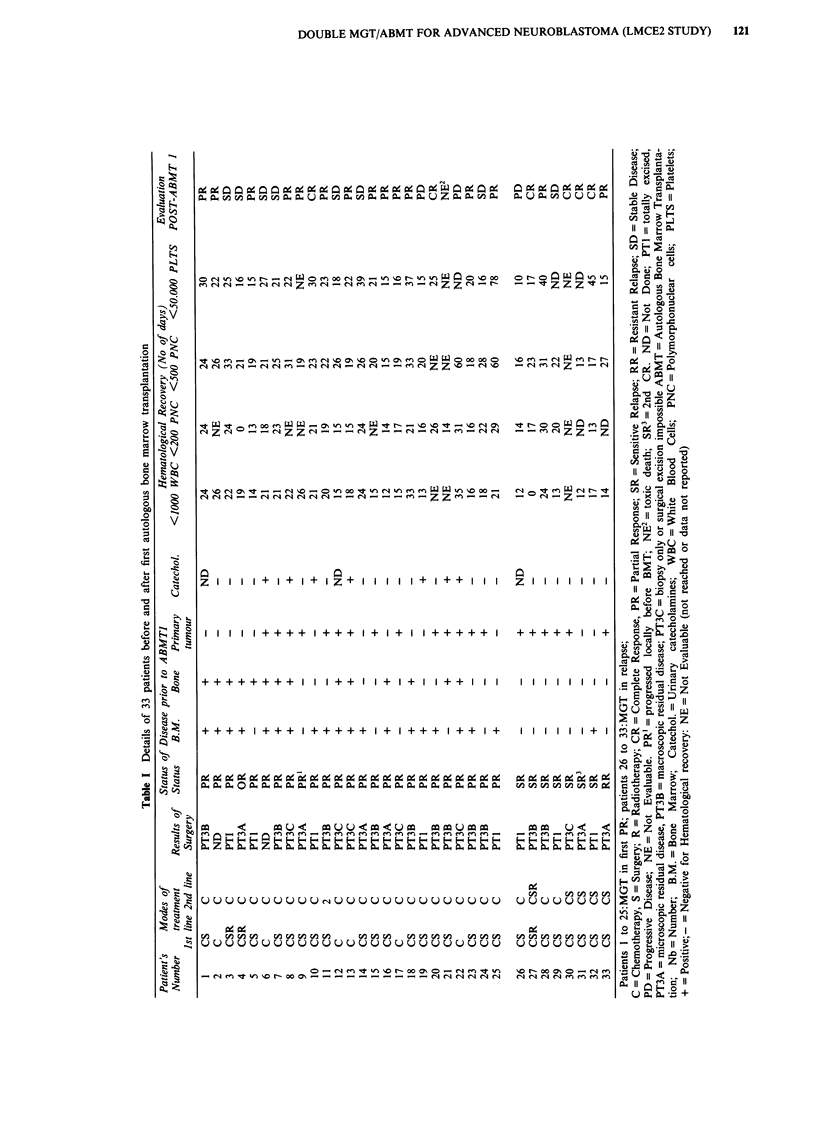

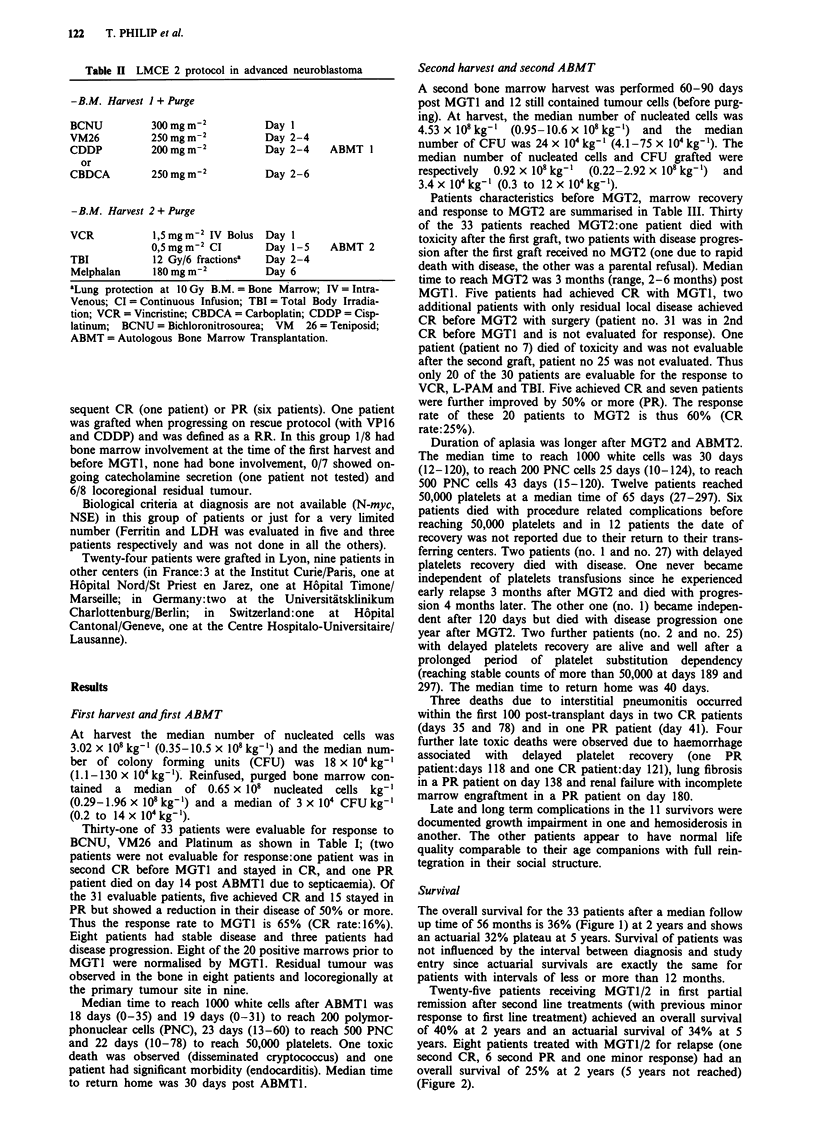

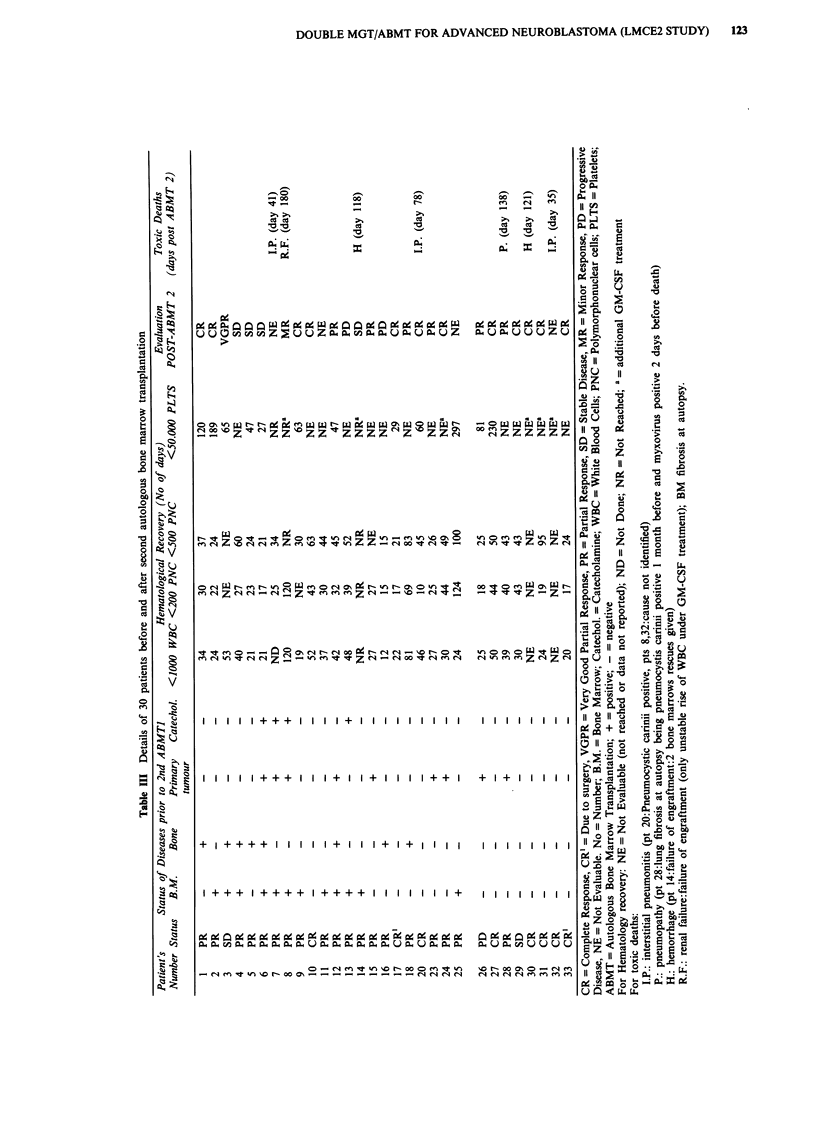

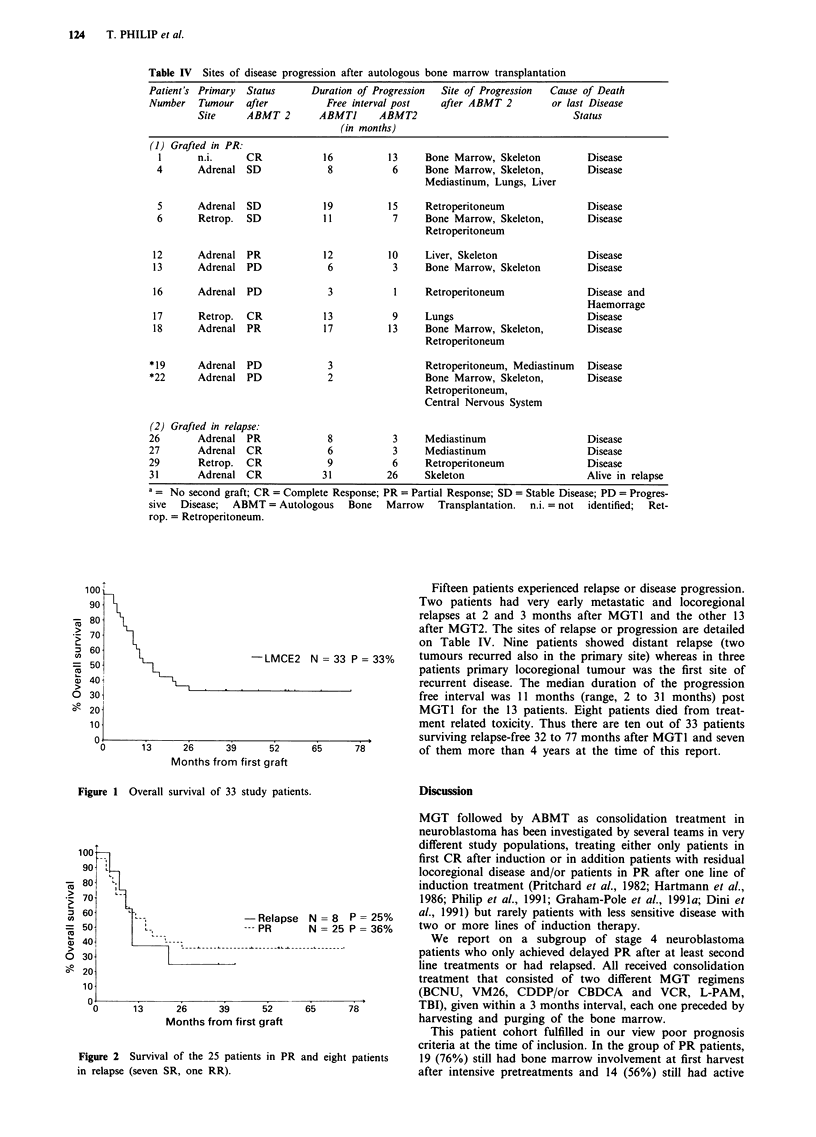

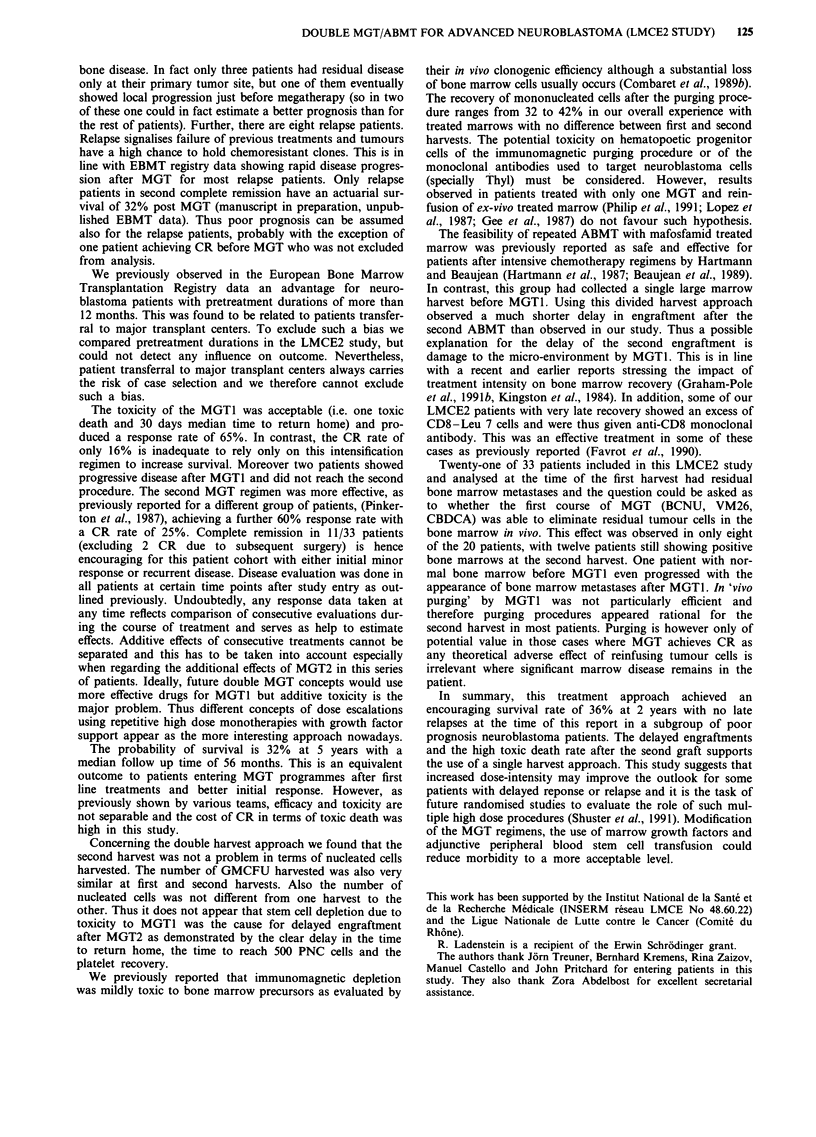

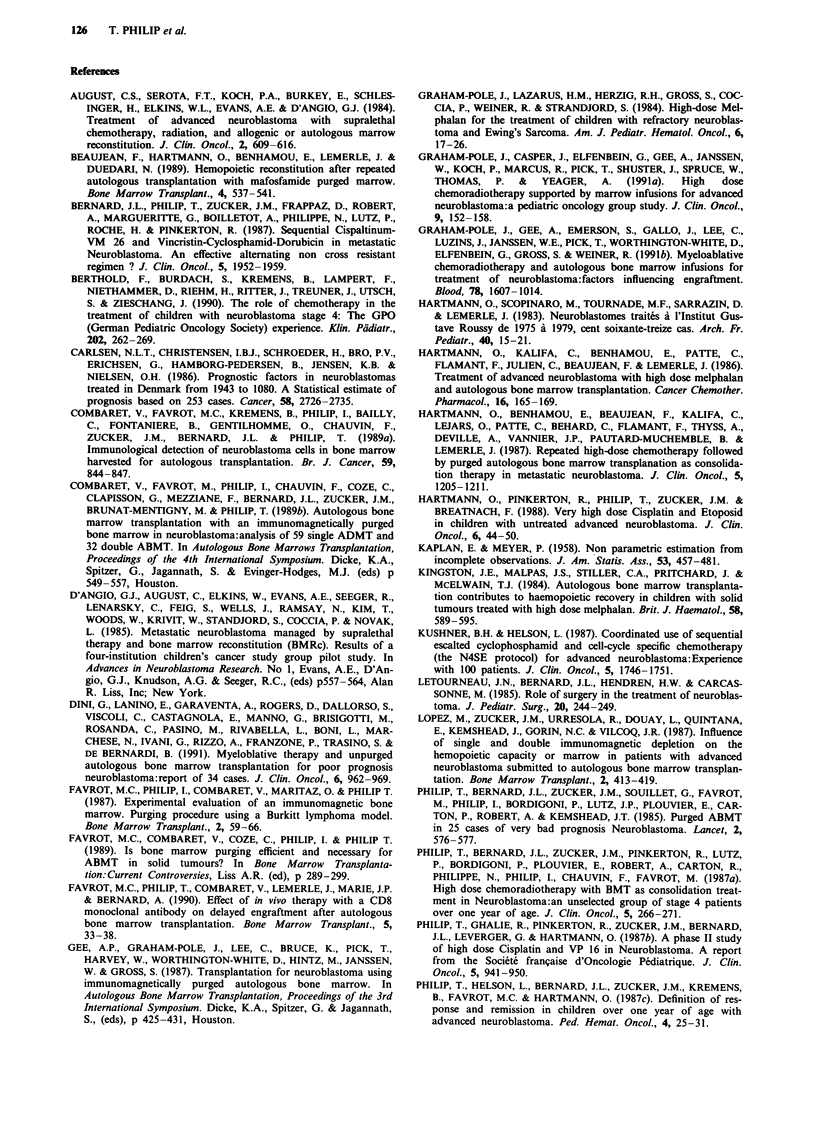

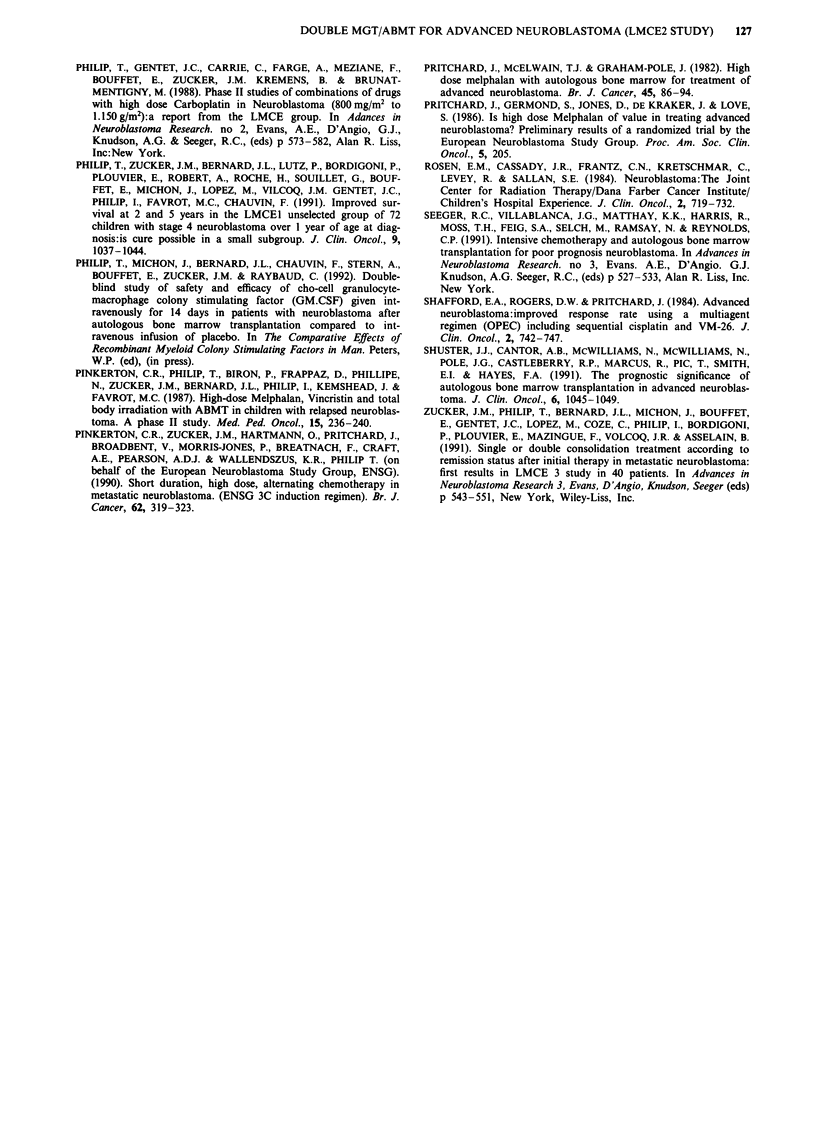

